# The impact of a human papillomavirus (HPV) vaccination campaign on routine primary health service provision and health workers in Tanzania: a controlled before and after study

**DOI:** 10.1186/s12913-018-2976-2

**Published:** 2018-03-12

**Authors:** Katherine E. Gallagher, Tusajigwe Erio, Kathy Baisley, Shelley Lees, Deborah Watson-Jones

**Affiliations:** 1 London School of Hygiene and Tropical Medicine, Clinical Research Department, Keppel St, London, WC1E 7HT UK; 2 Mwanza Intervention Trials Unit, National Institute for Medical Research, PO Box 11936, Mwanza, Tanzania; 3 London School of Hygiene and Tropical Medicine, Department of Infectious Disease Epidemiology, Keppel St, London, WC1E 7HT UK; 4 London School of Hygiene and Tropical Medicine, Department of Global Health and Development, 15-17 Tavistock Place, London, WC1H 9SH UK

**Keywords:** Human papillomavirus, Vaccination, Health systems, Human resources for health, Tanzania

## Abstract

**Background:**

The burden of cervical cancer and shortage of screening services in Tanzania confers an urgent need for human papillomavirus (HPV) vaccination. However, the sustainability and impact of another new vaccine campaign in an under-resourced health system requires consideration. We aimed to determine the impact of the government’s school-based HPV vaccine campaign in Kilimanjaro region on the provision of routine primary health services and staff workload.

**Methods:**

Data on daily numbers of consultations were collected from health facility register books in 63 dispensaries and health centres in North-West Tanzania for 20 weeks in 2014. Changes in outpatient, antenatal care (ANC), family planning (FP) and immunisation service activity levels before, during and after the two HPV vaccination campaigns in 2014 in 30 facilities within Kilimanjaro region (‘intervention facilities’) were compared with changes in activity levels in 33 facilities in Arusha region (‘controls’). Qualitative interviews were conducted with health workers in Kilimanjaro region who delivered HPV vaccination and those who remained at the facility during in-school HPV vaccine delivery to explore perceptions of workload and capacity.

**Results:**

Health facility activity levels were low and very variable in both regions. Controlling for district, facility type, catchment population, clinical staff per 1000 catchment population and the timing of other campaigns, no evidence of a decrease in consultations at the health facility during HPV vaccination week was found across outpatient, ANC, routine immunisation and FP services. However, compared to the average week before and after the campaign, health workers reported longer working hours and patient waiting times, feeling over-stretched and performing duties outside their normal roles whilst colleagues were absent from the facility conducting the HPV vaccine campaign.

**Conclusion:**

Qualitative interviews with health workers revealed that staff absence from the health facility is common for a number of reasons, including vaccination campaigns. Health workers perceived that the absence of their colleagues increased the workload at the health facility. The numbers of consultations for each service on ‘normal days’ were low and highly variable and there was no clear detrimental effect of the HPV vaccination campaign on routine health service activity.

**Electronic supplementary material:**

The online version of this article (10.1186/s12913-018-2976-2) contains supplementary material, which is available to authorized users.

## Background

Resource-poor countries carry over 85% of the cervical cancer disease burden, the third most common cancer in women worldwide [1–3]. Screening services are limited across sub-Saharan Africa [3]. There are two widely licensed, safe and efficacious human papillomavirus (HPV) vaccines targeting two HPV types that cause 70% of cervical cancer, HPV 16 and 18 [4]. A 9-valent vaccine targeting an additional 5 oncogenic HPV genotypes has been licensed in the USA and Europe [4–6]. HPV vaccine delivery is recommended by the World Health Organization (WHO) for 9–13 year old girls in a two-dose schedule [7]. In 2012 Gavi, the Vaccine Alliance, announced funding for HPV vaccine pilot ‘demonstration projects’ or national programmes in low and middle income countries (LAMICs) [8]. The majority of HPV vaccine delivery in LAMICs to date has used campaign-type approaches; nurses from health facilities visit sites in their catchment area for a set number of days to deliver each dose to a target population that is often not routinely targeted for vaccines [7, 9–14].

Introducing a multi-dose vaccine such as the HPV vaccine to a novel target population that requires outreach activities and careful community mobilisation could potentially stress under-resourced health systems and affect the delivery of other services [14–16]. Four studies in LAMIC examining the impact of new vaccine introductions on health systems published limited information on the impact on routine healthcare activities [17]. However, several recent studies in The Gambia and Cameroon have suggested that essential maternal and child health services and basic outpatient care have been affected during childhood vaccine campaigns (e.g. polio, measles) [18–22].

In 2014, the Tanzanian Ministry of Health and Social Welfare (MoHSW) began a Gavi-supported HPV vaccine demonstration project using school-based delivery in Kilimanjaro region, northern Tanzania [8]. Tanzania had only 43.6% of the WHO recommended number of health workers required to deliver a health service of minimum quality, with one nurse or midwife per 2300 people and 1 doctor per 32,300 people [23, 24]. Out-of-station activities by health workers have been shown to lead to task-shifting and increased work schedules for those staff remaining in the health facilities [25]. The aim of this study was to determine the impact of HPV vaccine delivery in Kilimanjaro region on the provision of routine primary health care services and health facility staff workload.

## Methods

### Study design

A retrospective, controlled analysis of health facility data before, during and after the HPV vaccination campaign was completed to assess the impact of the campaign week on levels of routine service provision at the health facilities. Key informant (KI) interviews with health workers in facilities involved in HPV vaccine delivery explored the perceived impact of the vaccine introduction on the facility and workload.

### The HPV vaccine demonstration project

HPV vaccine was delivered in Kilimanjaro region only by the MoHSW between 3-9th May 2014 (dose 1) and between 1-7th November 2014 (dose 2), by health facility nurses in primary schools within their facility catchment area. Vaccine was offered to all schoolgirls who were in grade 4 and who were aged 9 years or older. Out-of-school girls who were aged 9 years were informed by community mobilisation activities that they were eligible to receive the vaccine in health facilities.

### Study site selection

All government health facilities in Kilimanjaro region were involved in the demonstration project. Kilimanjaro region was considered the ‘intervention area’ and neighbouring Arusha region was the ‘control area’. Of the neighbouring regions, Arusha region was chosen because it was considered to be the most similar to Kilimanjaro region in geographic and socio-economic indicators [26].

Two districts were selected from each region to participate in the study so that intervention and control areas were similar with respect to population size and urbanisation (Table 1). For the quantitative analysis, 33 control and 30 intervention health facilities (government health centres or dispensaries) were randomly selected, proportional to the total number of health facilities in each study district. This ensured data from at least 30 intervention and control facilities were available for each activity indicator. Assuming a mean of 20 ante-natal care visits per week in the control facilities and a standard deviation of 10, 30 facilities in each region conferred 80% power to detect a 36% reduction in the number of visits in the intervention clinics in the week of the vaccination campaign.

**Table 1:** Districts and facilities selected for data collection

Region	Selected districts	Description and total population^a^	Total facilities^b^	No. selected facilities quantitative data collection	No. selected facilities qualitative interviews^c^
Kilimanjaro	Moshi District Council	Semi-urban 466,737	50	17 (4 Health centres, 13 dispensaries)	5
	Hai	Rural 210,533	38	13 (4 health centres, 9 dispensaries)	7
Arusha^d^	Arusha District Council	Semi-urban 323,198	27	10 (5 health centres, 5 dispensaries)	0
	Meru	Rural 268,144	55	23 (5 health centres, 18 dispensaries)	0

^a^The United Republic of Tanzania 2012 Population and Housing Census General Report. Dar Es Salaam, Tanzania: National Bureau of Statistics Ministry of Finance Dar Es Salaam & Office of the Chief Government Statistician Finance Economy and Development Planning Zanzibar, 2012

^b^Total number of government health centres or dispensaries in each district obtained from district Ministry of Health and Social Welfare officials

^c^The selection of facilities for qualitative data collection was determined by availability of interviewees

^d^33 health facilities were selected in Arusha region to ensure at least 30 facilities contributed data on each service; register books were missing from 4 facilities for some services

KI interviews with health workers were completed in a random sub-set of 12 of the 30 health facilities in Kilimanjaro region (Table 1). Interviewees were selected on the day the health facility was visited.

### Data collection and analysis

Weekly counts of consultations for four routine activities were retrospectively collected from facility register books: outpatient care among children under 5 years (OPD), Expanded Program on Immunization (EPI) visits, first antenatal care (ANC) visits and family planning (FP) consultations. These were well-defined activities that were routinely documented in separate register books. For each HPV vaccine dose and the equivalent calendar periods in control facilities, data were collected for 10 weeks, including the 4 weeks preceding and five weeks after the week of HPV vaccine delivery. Two data collectors entered data into Excel. The first author (KEG) validated data entry accuracy for 5% of register books. The most senior facility staff member available provided information on number and cadre of staff, dates of training, other campaigns and the organisation of HPV vaccination teams.

**Fig. 1:**
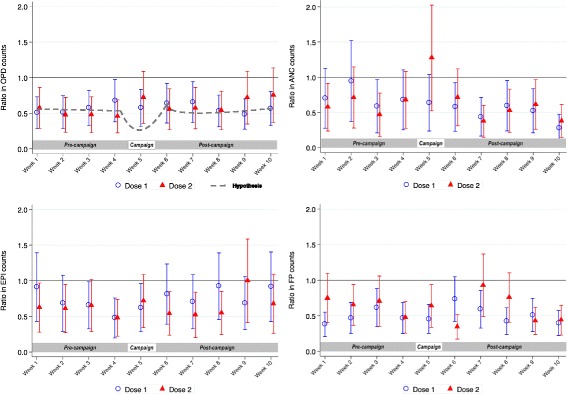
Ratios, and 95% confidence intervals, of mean counts of the four activity indicators in intervention facilities compared with control facilities in the weeks before, during and after HPV vaccine delivery^1.1^Estimatated by the negative binomial regression model adjusted for district, facility type (dispensary or health centre), catchment population, total clinical staff per 1000 catchment population per facility, timing of other campaigns

The effect of the HPV vaccine campaign week on each activity indicator was assessed using negative binomial regression in Stata 13.0 (StataCorp; TX, USA), with random effects to account for the correlation of repeated observations within facilities. Models were fitted to the weekly counts of each activity with the clinic’s catchment population as an offset. Separate analyses were done on the second HPV dose. Initial models contained fixed effects for region (intervention/control), time period (pre-campaign, campaign, post-campaign), and a region - time period interaction. Adjusted models included terms for catchment population, rural/urban location, facility type (centre/dispensary), the number of clinical staff per 1000 catchment population, and whether other campaigns or training were conducted in the period of interest, all identified a-priori as potential confounders. We hypothesised that, if the HPV vaccine campaign had an impact on activity, the difference between the intervention and control facilities would differ by time period (i.e. a significant region – time period interaction), and there would be a pattern in the differences in activity between the weeks before, during and after the campaign (Fig. 1).

In Kilimanjaro region, 10 KI who delivered HPV vaccine at schools, and nine who remained at the facility during HPV vaccination activities were interviewed. After informed consent, a Tanzanian female research assistant conducted interviews in Swahili that included questions on health workers’ perceptions of routine workload, staff capacity and the impact of the HPV vaccine delivery (Additional files 1 and 2: Figures S3 and S4). Interview data were coded using Nvivo 10 software (QSR International Pty Ltd. Cardigan, UK) according to a pre-designed framework of research questions, new codes were created as they arose. The coding was conducted by KEG and reviewed by a senior social scientist, SL. Convergent mixed methods analysis gave equal weight to each component [27–29].

## Results

Across the 63 facilities, a mean of 7 full-time clinical staff were registered to work per facility (e.g. doctors, nurses, medical attendants; range 1–61). Intervention and control facilities had around 1 clinical staff per 1000 population. The mean catchment population per facility was higher in Arusha compared to Kilimanjaro (Table 2).

**Table 2:** Characteristics of health facilities included in the study

Selected facilities included in the study	Control region (Arusha; *n* = 33)	Intervention region (Kilimanjaro; *n* = 30)
Health centres, dispensaries	10, 23	8, 22
Total catchment population (adults and children)	416,606	217,813
Facility characteristics:		
Mean catchment population per facility (s.d.)	12,624 (18,423)	7260 (4906)
Range in catchment population per facility	300–75,000	956–22,108
Mean number of clinical staff per facility (s.d.)	6.9 (11.1)	7.0 (5.4)
Range in total number of clinical staff per facility	1–61	2–19
Mean number of primary schools per facility (s.d.)	2.79 (1.54)	4.20 (2.93)
Range in number of primary schools per facility	1–7	1–14
Mean number of clinical doctors per facility per 1000 catchment population (s.d)	0.27 (0.43)	0.25 (0.23)
Mean number of registered nurses per facility per 1000 catchment population (s.d)	0.25 (0.49)	0.31 (0.29)
Mean number of enrolled nurses per facility per 1000 catchment population (s.d.)	0.46 (0.69)	0.36 (1.57)
Mean number of medical attendants per facility per 1000 catchment population (s.d.)	0.14 (0.10)	0.56 (0.43)
Mean total number of clinical staff per facility per 1000 population (s.d.)	0.99 (1.38)	1.10 (0.63)
Mean number of days spent delivering HPV vaccine dose 1 (s.d; range)	NA	2.83 (1.12; 1–5)
Mean number of days spent delivering HPV vaccine dose 2 (s.d; range)	NA	2.90 (1.45; 1–6)
Mean number of staff on HPV vaccination team doses 1 and 2 (s.d; range)	NA	2.53 (1.15; 1–6)
Proportion of total clinical staff workforce involved in HPV vaccination outreach team per facility (s.d)	NA	0.50 (0.25; 0.12–1.2)
Mean number of girls targeted in school per facility for dose 1 (s.d; range)	NA	65.6 (50.0; 8–242)
Mean number of out of school girls targeted for HPV vaccine per facility for dose 1 (s.d; range)	NA	1.2 (3.1; 0–15)
Mean total number of girls targeted for dose 1 per facility (s.d; range)	NA	66.8 (50.2; 8–242)
Mean number of doses delivered per facility dose 1 (s.d; range)	NA	64.6 (44.3; 9–205)
Mean number of doses delivered per facility dose 2 (s.d; range)	NA	63.3 (47.5; 9–196)
Mean coverage per facility dose 1 (s.d; range)	NA	103% (28.2; 85–238)
Mean coverage per facility dose 2 (s.d; range)	NA	94.8% (9.8; 70–117%)

*NA* not applicable

In the 30 Kilimanjaro facilities, on average, vaccination teams of 3 clinical staff took 3 days to visit 4 primary schools in their catchment area to deliver each vaccine dose (range 1–6 days; Table 2). The mean proportion of the workforce absent from the facility to deliver the vaccine was 50% (range 10%–100%). In 12 (40%) facilities, 70% or more of the workforce left the facility to deliver the vaccine. Administrative data of the target population of eligible girls and the number of doses delivered indicated 94.8% mean dose-2 coverage (range across facilities 70–117%; Table 2).

### Impact of HPV vaccine delivery on routine services

The average number of consultations per week for under-5 OPD, ANC, EPI and FP services varied considerably across all time points within facilities in both intervention and control sites. The under-5 OPD was the busiest service, with a range of 13–21 consultations per week across pre- and post-HPV vaccine campaign periods for both doses. There was a range of 1 to 8 first ANC consultations, 2 to 10 EPI consultations and 8–24 FP consultations per week. Mean counts of OPD, ANC, EPI and FP visits per week were lower in the intervention facilities than in the controls during the observation periods for both doses (Tables 3 and 4).

**Table 3:** Impact of the HPV vaccine campaign (dose 1 delivery) on routine services

Dose 1 weeks	Number of weeks control/intervention	Control facilities mean consults per week (s.d.)	Intervention facilities mean consults per week (s.d.)	Unadjusted RR *p*-value for interaction	Adjusted RR* *p*-value for interaction
Outpatient visits in children under 5 years				0.900	0.978
Pre-Campaign weeks	116/116	16.7 (13.5)	15.3 (10.7)	0.73 (0.52–1.03)	0.62 (0.43–0.90)
Campaign weeks	28/29	13.4 (10.6)	14.9 (12.6)	0.70 (0.46–1.05)	0.63 (0.41–0.98)
Post-campaign weeks	140/145	17.1 (10.5)	17.8 (12.1)	0.72 (0.51–1.00)	0.63 (0.44–0.90)
%change campaign vs pre-campaign		*−19.8%*	*−2.6%*		
% change post- vs. pre-campaign		*+ 2.4%*	*+ 16.3%*		
First antenatal care visits				0.018	0.026
Pre-Campaign weeks	116/116	3.2 (4.7)	2.1 (2.2)	1.06 (0.70–1.58)	0.73 (0.45–1.17)
Campaign weeks	29/29	3.1 (3.6)	1.6 (1.7)	0.84 (0.48–1.48)	0.65 (0.34–1.21)
Post-campaign weeks	145/145	4.3 (7.3)	2.0 (2.4)	0.72 (0.49–1.06)	0.48 (0.30–0.77)
%change campaign vs pre-campaign		*−3.1%*	*−23.8%*		
% change post- vs. pre-campaign		*+ 34.3%*	*−4.8%*		
Routine Immunisation visits				0.200	0.341
Pre-Campaign weeks	120/120	7.7 (20.6)	2.4 (2.4)	0.87 (0.62–1.23)	0.66 (0.43–1.01)
Campaign weeks	30/30	7.9 (24.0)	2.3 (2.6)	0.68 (0.42–1.11)	0.60 (0.35–1.04)
Post-campaign weeks	150/150	7.4 (21.0)	2.8 (2.6)	0.98 (0.70–1.36)	0.78 (0.52–1.17)
%change campaign vs pre-campaign		*+ 2.6%*	*−4.2%*		
% change post- vs. pre-campaign		*−3.9%*	*+ 14.2%*		
Family Planning consultations				0.792	0.631
Pre-Campaign weeks	124/120	12.4 (14.7)	9.8 (8.2)	1.11 (0.85–1.46)	0.49 (0.34–0.69)
Campaign weeks	31/30	14.6 (18.7)	11.4 (15.9)	1.01 (0.69–1.49)	0.46 (0.30–0.72)
Post-campaign weeks	155/150	16.8 (32.5)	13.8 (15.7)	1.14 (0.87–1.49)	0.53 (0.38–0.75)
%change campaign vs pre-campaign		*+ 17.7%*	*+ 16.3%*		
% change post- vs. pre-campaign		*+ 35.5%*	*+ 40.8%*		

*RR the ratio in the mean number of consultations for each service in the intervention and control facilities in each week, adjusted for district, facility type (dispensary or health center), catchment population, total clinical staff per 1000 catchment population per facility, timing of other campaigns. *P*-values for interaction test the hypothesis that the effect of time period in relation to the HPV vaccination campaign on activity (counts of consultations) differs between the intervention and control facilities i.e. the campaign weeks have an effect on activity in intervention facilities but not on control facilities

Italicised data indicates the relative change in the number of mean consultations per week, within each region, between the specified time-periods around the vaccination campaign

**Table 4:** Impact of the HPV vaccine campaign (dose 2 delivery) on routine services

Dose 2 weeks	Number of weeks control/intervention	Control facilities mean consults per week (s.d.)	Intervention facilities mean consults per week (s.d.)	Unadjusted RR *p*-value for interaction	Adjusted RR* *p*-value for interaction
Outpatient visits in children under 5 years				< 0.001	0.002
Pre-Campaign weeks	120/120	19.1 (20.7)	13.3 (10.1)	1.37 (0.97–1.91)	0.50 (0.32–0.79)
Campaign weeks	32/30	15.6 (19.3)	13.0 (9.7)	1.69 (1.14–2.50)	0.72 (0.44–1.19)
Post-campaign weeks	160/146	16.8 (15.0)	14.6 (10.0)	1.92 (1.38–2.69)	0.62 (0.40–0.98)
%change campaign vs pre-campaign		*−18.3%*	*−2.3%*		
% change post- vs. pre-campaign		*−12.0%*	*+ 9.8%*		
First antenatal care visits				< 0.001	< 0.001
Pre-Campaign weeks	116/116	5.2 (14.0)	1.8 (1.9)	0.81 (0.54–1.21)	0.59 (0.37–0.96)
Campaign weeks	30/29	4.1 (7.4)	3.0 (3.6)	1.59 (0.97–2.62)	1.23 (0.68–2.22)
Post-campaign weeks	150/145	7.9 (24.9)	2.0 (2.5)	0.61 (0.41–0.91)	0.50 (0.32–0.80)
%change campaign vs pre-campaign		*−21.2%*	*+ 66.7%*		
% change post- vs. pre-campaign		*+ 51.9%*	*+ 11.1%*		
Routine Immunisation visits				0.585	0.659
Pre-Campaign weeks	124/120	7.9 (19.8)	2.3 (2.4)	0.82 (0.59–1.14)	0.57 (0.38–0.86)
Campaign weeks	31/30	9.1 (21.0)	2.8 (3.0)	0.93 (0.59–1.46)	0.70 (0.42–1.19)
Post-campaign weeks	155/150	6.4 (15.1)	2.2 (2.6)	0.94 (0.68–1.31)	0.62 (0.41–0.93)
%change campaign vs pre-campaign		*+ 15.2%*	*+ 21.7%*		
% change post- vs. pre-campaign		*−19.0%*	*−4.3%*		
Family Planning consultations				0.424	0.499
Pre-Campaign weeks	124/120	13.2 (18.8)	11.3 (11.5)	1.06 (0.80–1.40)	0.58 (0.42–0.79)
Campaign weeks	31/30	10.5 (10.1)	9.1 (7.7)	1.26 (0.83–1.91)	0.60 (0.37–0.95)
Post-campaign weeks	155/150	17.2 (21.0)	13.9 (19.8)	0.97 (0.75–1.27)	0.50 (0.36–0.69)
%change campaign vs pre-campaign		*−20.5%*	*−19.5%*		
% change post- vs. pre-campaign		*+ 30.3%*	*+ 23.0%*		

*RR the ratio in the mean number of consultations for each service in the intervention and control facilities in each week, adjusted for district, facility type (dispensary or health center), catchment population, total clinical staff per 1000 catchment population per facility, timing of other campaigns. *P*-values for interaction test the hypothesis that the effect of time period in relation to the HPV vaccination campaign on activity (counts of consultations) differs between the intervention and control facilities i.e. the campaign week has an effect on activity in intervention facilities but not on control facilities

Italicised data indicates the relative change in the number of mean consultations per week, within each region, between the specified time-periods around the vaccination campaign

In adjusted analyses, there was some evidence of a difference between intervention and control facilities in the weekly average number of ANC consultations before, during and after the dose 1 campaign (*p* value for interaction =0.026). However, there was no indication of a pattern over time or any evidence that the difference between intervention and control facility activity was greater during the HPV vaccine campaign week (Table 3; Fig. 1).

In the periods around the second dose, there was strong evidence that the effect of the time period on OPD and ANC visits differed between the intervention and control facilities (*p* values for interaction = 0.002 and < 0.001, respectively). However, the difference between activity in intervention and control facilities decreased in the campaign week (the adjusted ratio of activity between intervention and control facilities became closer to 1.0) i.e. the evidence of an interaction was in the opposite direction to our hypothesis (Table 4; Fig. 1).

**Fig. 2:**
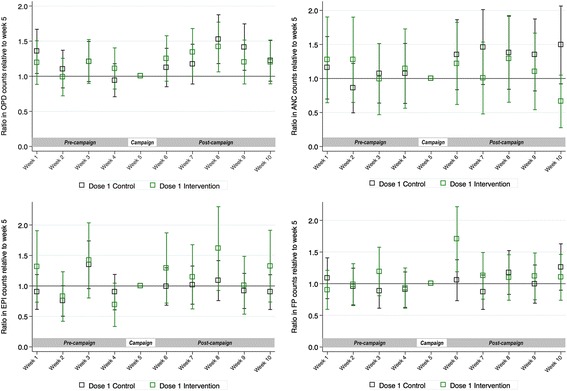
Ratios, and 95% confidence intervals, of mean counts of the four activity indicators during dose 1 delivery, comparing the weeks before and after the vaccine campaign with week 5 (HPV vaccine campaign week), or the equivalent time periods in control facilities^1^. ^1^Estimatated by the negative binomial regression model adjusted for district, facility type (dispensary or health centre), catchment population, total clinical staff per 1000 catchment population per facility, timing of other campaigns

When comparing the number of OPD, ANC, EPI and FP consultations over time within the intervention facilities alone, accounting for facility characteristics, there was no evidence of a decrease in consultations during the HPV vaccine campaign, relative to the 4 weeks pre- or post-campaign, in either the period around dose 1 or dose 2 (Fig. 2, Additional file 3: Figure S1). Exploratory analyses stratifying facilities into those where > 50% or ≤50% of the workforce was absent on vaccination days found no evidence of a decrease in average activity during the campaign week in either stratum (Additional file 4: Figure S2).

### Key informant interviews

The 19 KIs from 12 facilities were aged between 36 and 60 years old, with an average of 12 years’ experience working as health workers (range 1–31 years). There was overlap in different cadres’ reported roles and responsibilities (Table 5). An enrolled nurse, training to become a registered nurse, stated:

*“I perform all duties; sometimes when the doctor is not around I have to take over the doctor’s duties”. RV03*

Eight KIs from eight facilities reported that their routine workload was manageable and described having sufficient capacity to absorb more activities since the facility had adequate staffing levels and/or a small catchment population. Three KIs from three health centres indicated a heavy workload during ‘normal days’ with, insufficient staff, long waiting times, no time for rest breaks and working more than routine shifts. Staff absence was only cited as an issue by two KI:

*“….when another person is sick, you are all alone at the facility, overworking.” RV01*

*“During measles campaign days we become overloaded, there is a lot of work.” RV02*

During HPV vaccine delivery, some journeys to schools took 2 h. However, at schools, vaccinators could usually complete vaccination in half an hour to three hours per school, depending on the number of eligible girls. Mop-up activities increased the time that vaccinators spent away from the facility; up to four visits per school were conducted to reach girls who were initially absent from school, or refused vaccination:

*“Students may dodge from school, this led to the addition of more than those two or three planned days; they went for four days” RNV06*

Vaccinator KIs reported that sensitisation was more difficult and time-consuming for HPV vaccine compared to other vaccines because there was a low level of awareness about HPV and cervical cancer. However, it was clear that staff perceived the workload to deliver the HPV vaccine was less than that of other campaigns as it was a smaller target population:

*“For example, for that national measles rubella campaign almost all staff were out of the facility” RNV04*

Regarding the perceived impact of HPV vaccine introduction on routine activities, all nine KIs who stayed at their nine facilities during HPV vaccine activities stated that the campaign increased their workload and they had to employ strategies to cope with the staff shortage. These included task-shifting and working several hours longer each day (reported by 8 KIs), or deferring less urgent patients to the next day (1 KI). All but one KI reported that staff were not allowed to take leave during campaigns.

*“We have a person for every department but sometimes you find that two or three departments were attended by only a single person [during HPV vaccine delivery]. Sometimes you can be tired but just work because there is no way out” RNV01*

Four KI reported that patients had to wait substantially longer to be seen. Only one KI stated that there was no impact of HPV vaccine delivery on routine service provision, despite having to close the facility to deliver the vaccine in-schools, and attributed this to the fact that the facility had a small catchment population. No KI perceived that the HPV vaccine sensitisation activities in the community affected the uptake of other routine vaccines, or dissuaded patients from coming to the facility during the campaign week. KI reported that the accuracy of recording data in register books did not change during the campaigns.

**Table 5:** Key informant designation

Reported designation	HPV vaccinators	Non-vaccinators	Reported roles and responsibilities
Doctor	1	2	Supervision of all services and cleanliness, general administration of the facility, minor operations, referrals. Management of outpatient and reproductive and child health care.
Matron	0	1	Coordination of facility activities and colleagues, in-patient and out-patient care, MCH duties.
Registered nurse	2	2	Supervision of colleagues, provision of all services including delivery care, family planning, ANC, vaccinations, education, dispensing drugs.
Midwife	1	0	MCH, reproductive health.
Enrolled nurse	1	1	Vaccinations, under-5 outpatient services, dispensary, health education, MCH services, family planning, and outreach.
Auxiliary nurse	1	0	Assist every department.
Medical attendant/ MCH aider	4	3	Dispense drugs, dress wounds, vaccinations, assist ANC, delivery care, under-5 outpatient care, HIV VCT, and other reproductive health care, cleaning the facility.
Total	10	9	

*ANC* Antenatal care, *HIV VCT* human immunodeficiency virus voluntary counseling and testing, *MCH* Maternal and child health

KI were positive about the benefits of the HPV vaccine to the community and, despite the additional workload, many wanted to expand the target age group in order to protect more women. Overall, health workers involved in HPV vaccine activities supported school-based delivery, despite the transport issues and the increased workload because they could access more girls in schools and teachers could assist in sensitisation. They believed relying on facility-based delivery would lead to children absconding, or the distance between the facility and household being a barrier to vaccination.

## Discussion

Despite the human resource and health service constraints in Tanzania, at the current level of healthcare utilization, we found no evidence that the first year of an HPV vaccine school-based campaign in Kilimanjaro region affected the provision of consultations for routine outpatient, ANC, EPI and FP services during the HPV campaign week. A study in Rwanda also found no impact of HPV vaccine introduction on the provision of ANC services [30], although Rwanda has a relatively well-resourced health system in comparison to the rest of the region [12, 24]. There is some suggestion from our qualitative research that the quality of care provided at the facility during the HPV vaccine campaign could have been affected, with longer patient waiting times and staff performing some tasks outside their normal responsibilities.

The school-based ‘campaign’ delivery approach resulted in two to three health workers, half of an average facility’s workforce, leaving the facility to conduct outreach in schools for 3 days, twice in the year. The strategy led to very high vaccine coverage. Health workers mitigated the impact of staff absence on the provision of routine services at the facility despite the general shortage of health workers. The predominant mitigation strategies were to postpone annual leave, to work longer hours and to task-shift. It was common for enrolled nurses to perform the same tasks as registered (fully trained) nurses with or without supervision; this has been reported in previous studies [25, 31].

The size of the facility catchment populations and Tanzania’s crude birth rate of 38.1 births per 1000 population per year [26], leads us to expect at least double the number of ANC appointments than the two visits observed per week. The low level of utilisation and concentration of patient attendance in the morning hours is consistent with existing literature and could have contributed to our findings of a lack of impact of the campaign week on the number of routine consultations [31, 32].

The strengths of this study include the mixed methods design which allowed conclusions to be drawn with greater plausibility than would have been possible using either of the methods alone. The availability of data pre-, post-, and during the campaign week and the timing of this study should have limited recall bias [27]. The data collection team observed consistent use of register books during data collection activities. Qualitative data were analysed prior to quantitative data to avoid bias in the interpretation of transcripts. A range of experiences were captured at facilities with variable staffing levels and locations.

There were several limitations to this study. The extent of an impact on the quality of care, suggested by the qualitative research, was difficult to measure retrospectively. A substantial level of health worker absenteeism on ‘normal days’ has been reported in Tanzania [31]. Although KIs reported a reduction in health workers during campaign days, it is unclear how significant this impact was since patterns of staff absenteeism on ‘non-campaign’ days were not measured. Additionally, quantitative analysis could not control for absenteeism during ad-hoc outreach activities which were described in qualitative interviews but had no recorded dates.

We focused on four services at the clinic level. It is conceivable that HPV vaccine introduction could have affected a different selection of services at different facilities depending on the vaccinator nurses’ different roles. Kilimanjaro region may not be representative of the potential impact of vaccine introduction on health services nationwide since the region has a 10 per 10,000 ratio of health workers to population, compared to the national average of 7 per 10,000 and 4 per 10,000 in several regions [23, 25, 33].

## Conclusion

Qualitative interviews revealed that staff absence from the health facility is common for a number of reasons and health workers perceived that the absence of colleagues increased their workload. The numbers of consultations for each service on ‘normal’ (non-campaign) days were low and highly variable. We found no evidence that the absence of staff from the facility on HPV vaccine campaign weeks corresponded to a decrease in the number of consultations for routine services at the health facility when compared to non-campaign weeks, controlling for district, facility type, catchment population, clinical staff per 1000 catchment population and the timing of other formal campaigns. Further research on the impact of campaigns on health services is still necessary in order to determine their sustainability.

## Additional files

Additional file 1: **Figure S3.** Key informant interview topic guide for health workers involved in HPV vaccination activities. The interview topic guide used for key informant interviews with health workers who were involved in the HPV vaccination campaign. (PDF 30 kb)

Additional file 2: **Figure S4.** Key informant interview topic guide for health workers who remained at the health facility during the HPV vaccination campaign. The interview topic guide used for key informant interviews with health workers who remained at the health facility during the HPV vaccination campaign activities. (PDF 23 kb)

Additional file 3: **Figure S1.** Ratios, and 95% confidence intervals, of mean counts of the four activity indicators during dose 2 delivery, comparing the weeks before and after the vaccine campaign with week 5 (HPV vaccine campaign week), or the equivalent time periods in control facilities. Ratios, and 95% confidence intervals, of mean counts of the four activity indicators during dose 2 delivery, comparing the weeks before and after the vaccine campaign with week 5 (HPV vaccine campaign week), or the equivalent time periods in control facilities estimated by the negative binomial regression model adjusted for district, facility type (dispensary or health centre), catchment population, total clinical staff per 1000 catchment population per facility, timing of other campaigns. OPD: outpatient care consultations for children under 5 years; ANC: antenatal care consultations; EPI: routine immunisation consultations; FP: family planning consultations. (PDF 544 kb)

Additional file 4: **Figure S2.** Ratios, and 95% confidence intervals, of mean counts of the four activity indicators in intervention facilities compared with control facilities in the weeks before, during and after HPV vaccine delivery. Results are stratified by the proportion of the clinical workforce that was absent during the campaign. Ratios, and 95% confidence intervals, of mean counts of the four activity indicators in intervention facilities compared with control facilities in the weeks before, during and after HPV vaccine delivery. Results are stratified by the proportion of the clinical workforce that was absent during the campaign estimated by the negative binomial regression model adjusted for district, facility type (dispensary or health centre), catchment population, total clinical staff per 1000 catchment population per facility, timing of other campaigns. OPD: outpatient care consultations for children under 5 years; ANC: antenatal care consultations; EPI: routine immunisation consultations; FP: family planning consultations. (PDF 769 kb)

## Abbreviations

ANC: Ante-natal care
EPI: Expanded programme on immunisations
FP: Family planning
HPV: Human papillomavirus
KI: Key informant
LAMIC: Low and middle-income countries
MoHSW: The Tanzanian Ministry of Health and Social Welfare
OPD: Outpatient care
WHO: World Health Organization
