# Supplementary material for: The impact of a human papillomavirus (HPV) vaccination campaign on routine primary health service provision and health workers in Tanzania: a controlled before and after study
Source: BMC Health Serv Res. 2018 Mar 12;18:173. doi: 10.1186/s12913-018-2976-2 (PMC5848545; doi:10.1186/s12913-018-2976-2)
Supplement: Supplementary file 1 — Figure S3. Key informant interview topic guide for health workers involved in HPV vaccination activities. The interview topic guide used for key informant interviews with health workers who were involved in the HPV vaccination campaign. (PDF 30 kb) [file 12913_2018_2976_MOESM1_ESM.pdf]

Topic Guide: In-depth Interviews with health workers involved in HPV Vaccine implementation

A: Experience working at the facility

Now I am going to ask you questions about your experience on working at this facility

1. How long have you been working at this facility?
2. How many days per week do you work at this facility?
3. What are your roles at this facility?
4. Is your salary enough to support your family?
5. What do you think of the salary you are getting now? (Probe: Is it paid on time?)
6. Do you work elsewhere different from this facility?
7. Do you get incentives/allowance to complete certain extra services in the facility? (Probe: Do you live on extra per diems?)
8. Do you supplement your income another way? (Probe: If yes, what do you do)

B: Workload and staffing

Now I am going to ask you questions about workload at the facility in relation to staffing

9. What do you think of the workload at this facility? (Probe: is there too much work, an average workload or a low workload)
10. Is there enough staff in the workplace? (Probe: for the workload and available number of staff, do you get free time for tea and lunch? Do you feel stretched or are you able to absorb further work in the form of health campaigns?
11. In general do you feel you have adequate training and capacity for your roles at the health facility?)
12. How do you feedback to the district? Do you feel supported/ supervised/ autonomous in executing your work? Are there targets set by the district to fill? What are they? What do you do if you have not reached a target?
13. How do you take time off/leave? Are there times of year when it is easy to take time off/ annual leave? (Probe: Were you able to get time off/leave during HPV campaigns? If No, does this also happen during other campaigns like this one?)

C: HPV vaccine implementation

Now I am going to ask you questions about HPV vaccine implementation

14. Do you feel you got adequate training, and preparation for HPV vaccine delivery?
15. To what extent were health-workers involved in the planning of HPV vaccine delivery? (Probe: Choosing when it happened and how? Would you have planned it differently?)
16. What is your overall experience with HPV vaccine implementation and your perception towards the HPV vaccine?
17. To what extent people in the community know about HPV?
18. How well do you think the awareness and education campaign went before the vaccine delivery?
19. How much work was it to deliver the vaccine in schools last year (Probe on Opinions on the school site for vaccination?)

20. Do they perform other outreach activities in schools? How does the workload compare? Why does HPV vaccine implementation differ from these campaigns?

For HPV vaccination:

21. Was there just one visit per school, or did you have to make preparatory/ follow-up visits?
22. Did you get remuneration for the HPV vaccine delivery?
23. How long did it take for vaccine delivery in one school? How many schools did they visit? Did they have enough vaccine?
24. Was there a good supply of equipment and stock for the vaccine? How does the level of supply compare to the supply of equipment for other services and other campaigns?
25. Did HPV affect other services? Are the community now more or less engaged with the health facility? Did HPV vaccine affect other vaccines? Do they think it will affect other vaccines (awareness/ demand)?
26. What experience did they have collaborating with education officials?
27. What are the personal advantages or disadvantages of being involved in the vaccination?
28. Are there any particular successes or problems with the HPV vaccine implementation?
29. Did the experience differ when you delivered the second dose compared to the first?

We have come to the end of our interview, thank you for your time and collaboration. I would like to remind you that any information that you have shared with us will be kept highly confidential and it will only be used for the research purpose.
