# Supplementary material for: The impact of a human papillomavirus (HPV) vaccination campaign on routine primary health service provision and health workers in Tanzania: a controlled before and after study
Source: BMC Health Serv Res. 2018 Mar 12;18:173. doi: 10.1186/s12913-018-2976-2 (PMC5848545; doi:10.1186/s12913-018-2976-2)
Supplement: Supplementary file 2 — Figure S4. Key informant interview topic guide for health workers who remained at the health facility during the HPV vaccination campaign. The interview topic guide used for key informant interviews with health workers who remained at the health facility during the HPV vaccination campaign activities. (PDF 23 kb) [file 12913_2018_2976_MOESM2_ESM.pdf]

Topic Guide: In-depth Interviews with health workers at the facility not involved in HPV vaccination activities

A: Experience working at the facility

Now I am going to ask you questions about your experience on working at this facility

1. How long have you been working at this facility?
2. How many days per week do you work at this facility?
3. What are your roles at this facility?
4. Is your salary enough to support your family?
5. What do you think of the salary you are getting now? (Probe: Is it paid on time?)
6. Do you work elsewhere different from this facility?
7. Do you get incentives/allowance to complete certain extra services in the facility? (Probe: Do you live on extra per diems?)
8. Do you supplement your income another way? (Probe: If yes, what do you do)

B: Experience at the facility during HPV vaccine implementation

Now I am going to ask you questions about HPV vaccine implementation

9. What is your overall impression of HPV vaccine implementation for the last year (2014)? Would you want to be involved in delivering HPV vaccine if you could? Why/ why not?
10. How did the public respond to HPV vaccine?
11. How did it affect the people coming to the facility? (Probe: Were people aware of the existence of HPV vaccine and campaign?)
12. How did HPV vaccination activities affect the facility workload? Did it matter that one staff member was away? What did they do to overcome the gap?
13. Were staff able to take time off/leave during HPV vaccination delivery? If No, Why?
14. What is the supply of equipment/ drugs in the health centre like compared to regular vaccine supplies? Did HPV affect the supply of other vaccine equipment at the facility? Or was it timely or delayed than before?
15. Did vaccine storage equipment changed for HPV?
16. Did HPV vaccination activities affect uptake of other vaccines in the health facility? Did it increase awareness for other vaccine uptake in the facility? Or other services including other adolescent services in family planning or reproductive health?
17. What overall effect do you think HPV vaccine had on the health centre? (Probe: did it increase/decrease demand for services? Did it increase or decrease the health facility's ability to provide other routine services?
18. How did the experience at the health facility during HPV vaccination activities compare to periods of time during other vaccine campaigns e.g. the measles-rubella campaign?
19. Do you have anything that you would like to add concerning what we have discussed about HPV vaccine delivery?

We have come to the end of our interview, thank you for your time and collaboration. I would like to remind you that any information that you have shared with us will be kept highly confidential and it will only be used for the research purpose.
