# Supplementary material for: The impact of a human papillomavirus (HPV) vaccination campaign on routine primary health service provision and health workers in Tanzania: a controlled before and after study
Source: BMC Health Serv Res. 2018 Mar 12;18:173. doi: 10.1186/s12913-018-2976-2 (PMC5848545; doi:10.1186/s12913-018-2976-2)
Supplement: Supplementary file 3 — Figure S1. Ratios, and 95% confidence intervals, of mean counts of the four activity indicators during dose 2 delivery, comparing the weeks before and after the vaccine campaign with week 5 (HPV vaccine campaign week), or the equivalent time periods in control facilities. Ratios, and 95% confidence intervals, of mean counts of the four activity indicators during dose 2 delivery, comparing the weeks before and after the vaccine campaign with week 5 (HPV vaccine campaign week), or the equivalent time periods in control facilities estimated by the negative binomial regression model adjusted for district, facility type (dispensary or health centre), catchment population, total clinical staff per 1000 catchment population per facility, timing of other campaigns. OPD: outpatient care consultations for children under 5 years; ANC: antenatal care consultations; EPI: routine immunisation consultations; FP: family planning consultations. (PDF 544 kb) [file 12913_2018_2976_MOESM3_ESM.pdf]

Supplementary Figure 1: Ratios, and 95% confidence intervals, of mean counts of the four activity indicators during dose 2 delivery, comparing the weeks before and after the vaccine campaign with week 5 (HPV vaccine campaign week), or the equivalent time periods in control facilities[1].

[1]Estimatated by the negative binomial regression model adjusted for district, facility type (dispensary or health centre), catchment population, total clinical staff per 1000 catchment population per facility, timing of other campaigns. OPD: outpatient care consultations for children under 5 years; ANC: antenatal care consultations; EPI: routine immunisation consultations; FP: family planning consultations.

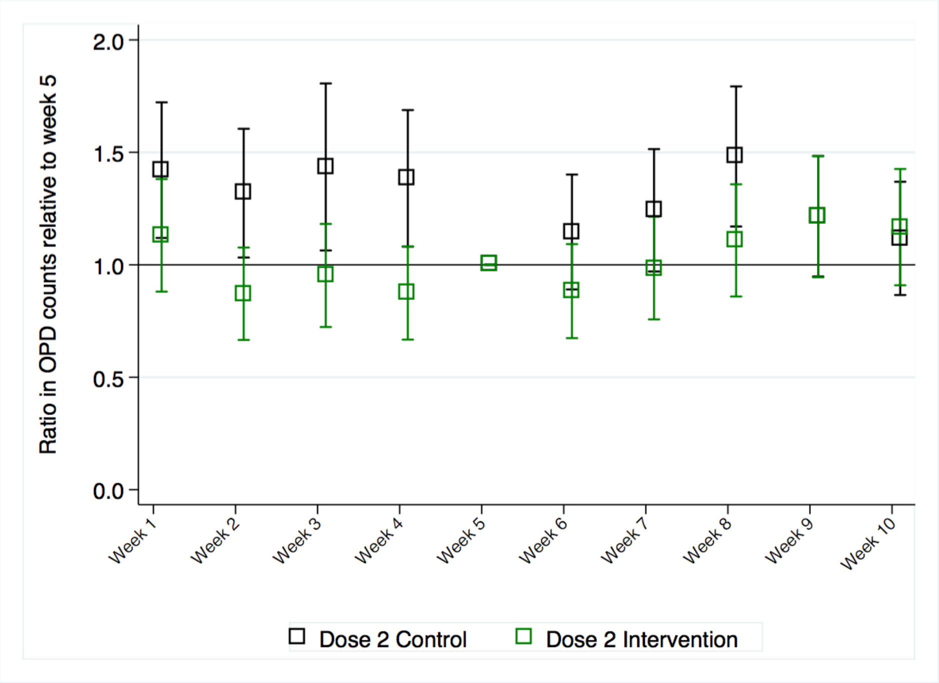

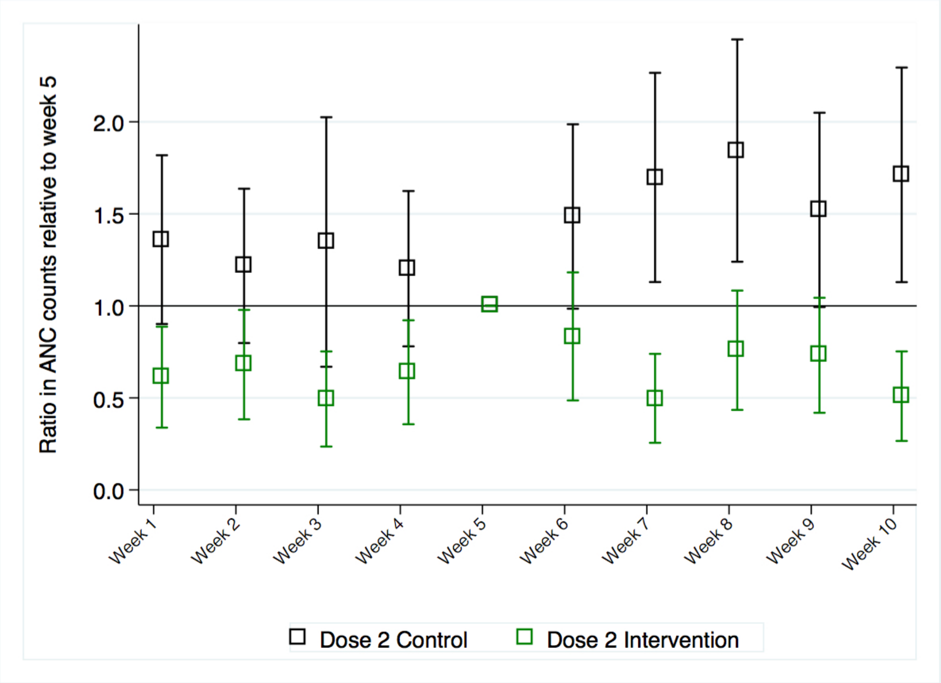

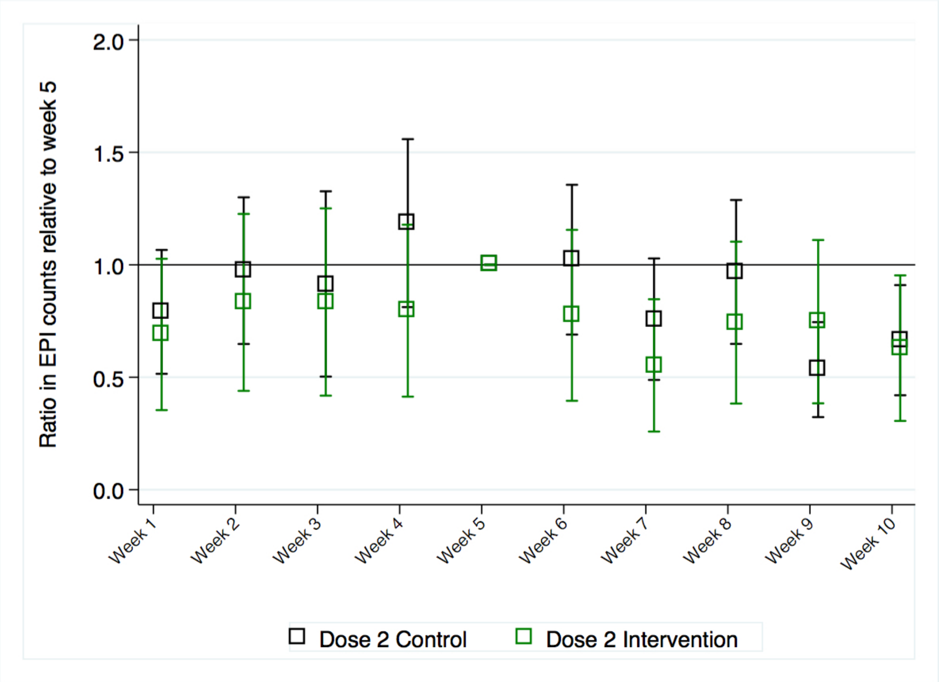

Ratio in EPI counts relative to week 5
2.0
1.5
1.0
0.5
0.0
Week 1
Week 2
Week 3
Week 4
Week 5
Week 6
Week 7
Week 8
Week 9
Week 10
Dose 2 Control
Dose 2 Intervention

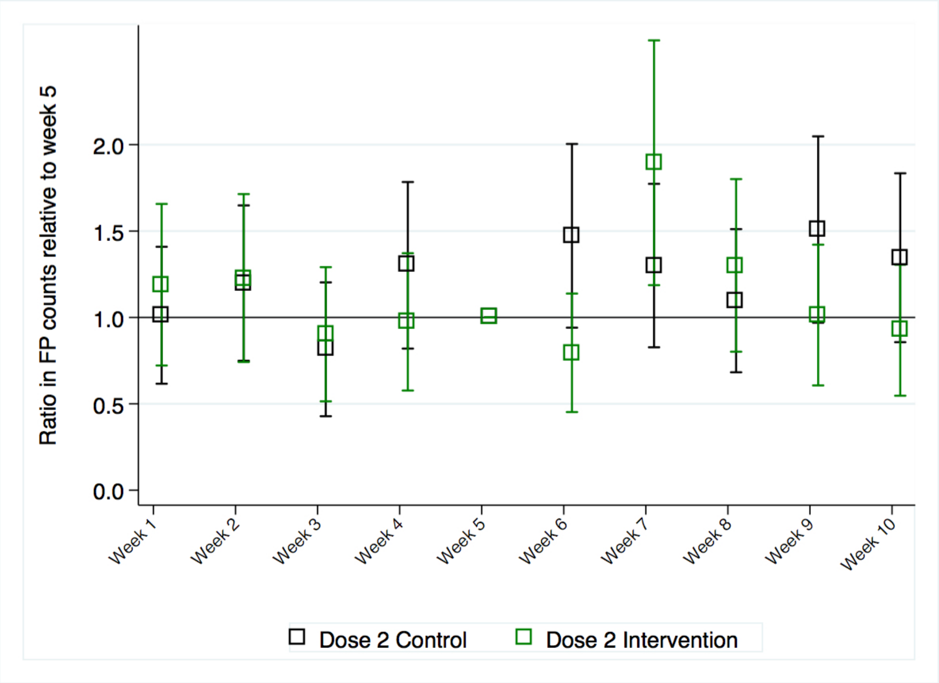

Ratio in FP counts relative to week 5
2.0
1.5
1.0
0.5
0.0
Week 1
Week 2
Week 3
Week 4
Week 5
Week 6
Week 7
Week 8
Week 9
Week 10
Dose 2 Control
Dose 2 Intervention
