# Supplementary material for: The impact of a human papillomavirus (HPV) vaccination campaign on routine primary health service provision and health workers in Tanzania: a controlled before and after study
Source: BMC Health Serv Res. 2018 Mar 12;18:173. doi: 10.1186/s12913-018-2976-2 (PMC5848545; doi:10.1186/s12913-018-2976-2)
Supplement: Supplementary file 4 — Figure S2. Ratios, and 95% confidence intervals, of mean counts of the four activity indicators in intervention facilities compared with control facilities in the weeks before, during and after HPV vaccine delivery. Results are stratified by the proportion of the clinical workforce that was absent during the campaign. Ratios, and 95% confidence intervals, of mean counts of the four activity indicators in intervention facilities compared with control facilities in the weeks before, during and after HPV vaccine delivery. Results are stratified by the proportion of the clinical workforce that was absent during the campaign estimated by the negative binomial regression model adjusted for district, facility type (dispensary or health centre), catchment population, total clinical staff per 1000 catchment population per facility, timing of other campaigns. OPD: outpatient care consultations for children under 5 years; ANC: antenatal care consultations; EPI: routine immunisation consultations; FP: family planning consultations. (PDF 769 kb) [file 12913_2018_2976_MOESM4_ESM.pdf]

Supplementary Figure 2. Ratios, and 95% confidence intervals, of mean counts of the four activity indicators in intervention facilities compared with control facilities in the weeks before, during and after HPV vaccine delivery. Results are stratified by the proportion of the clinical workforce that was absent during the campaign[1].

[1]Estimatated by the negative binomial regression model adjusted for district, facility type (dispensary or health centre), catchment population, total clinical staff per 1000 catchment population per facility, timing of other campaigns. OPD: outpatient care consultations for children under 5 years; ANC: antenatal care consultations; EPI: routine immunisation consultations; FP: family planning consultations.

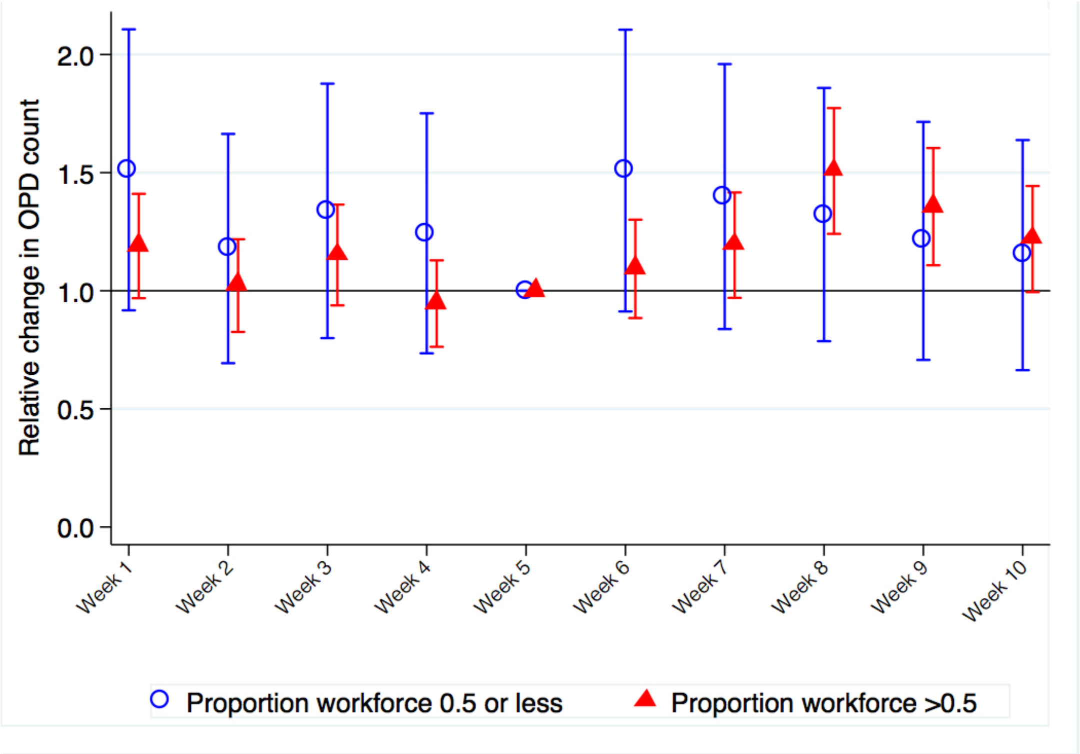

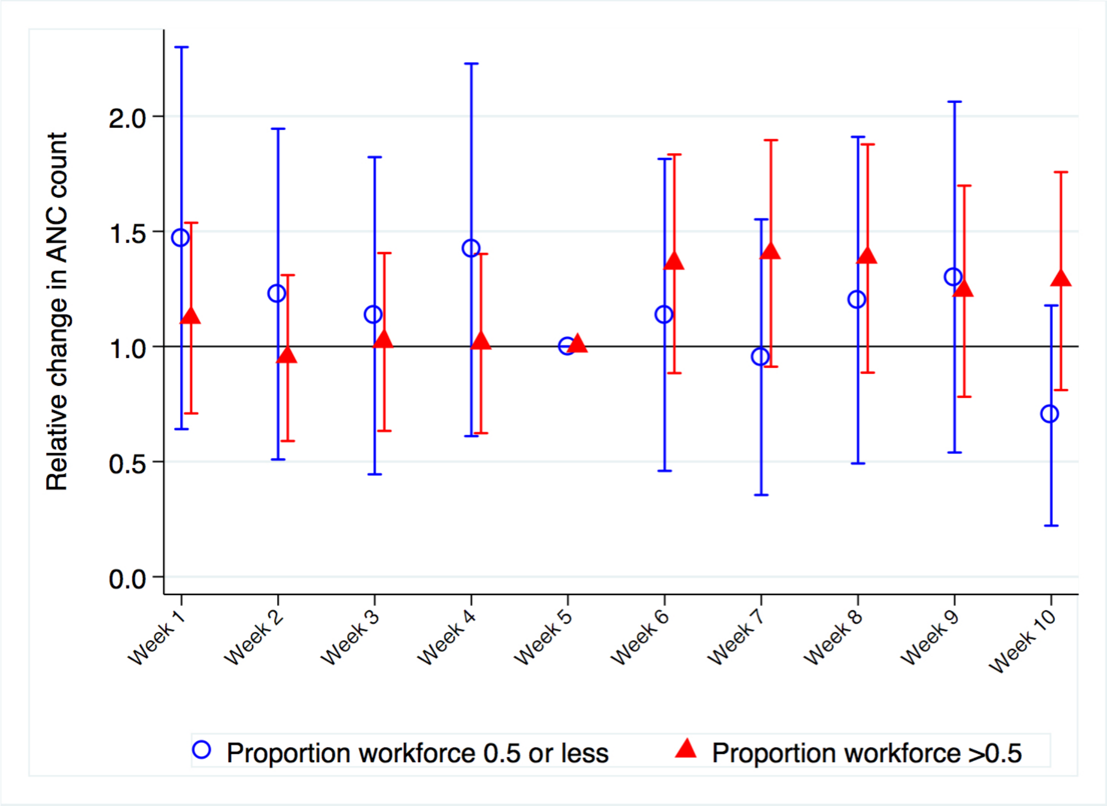

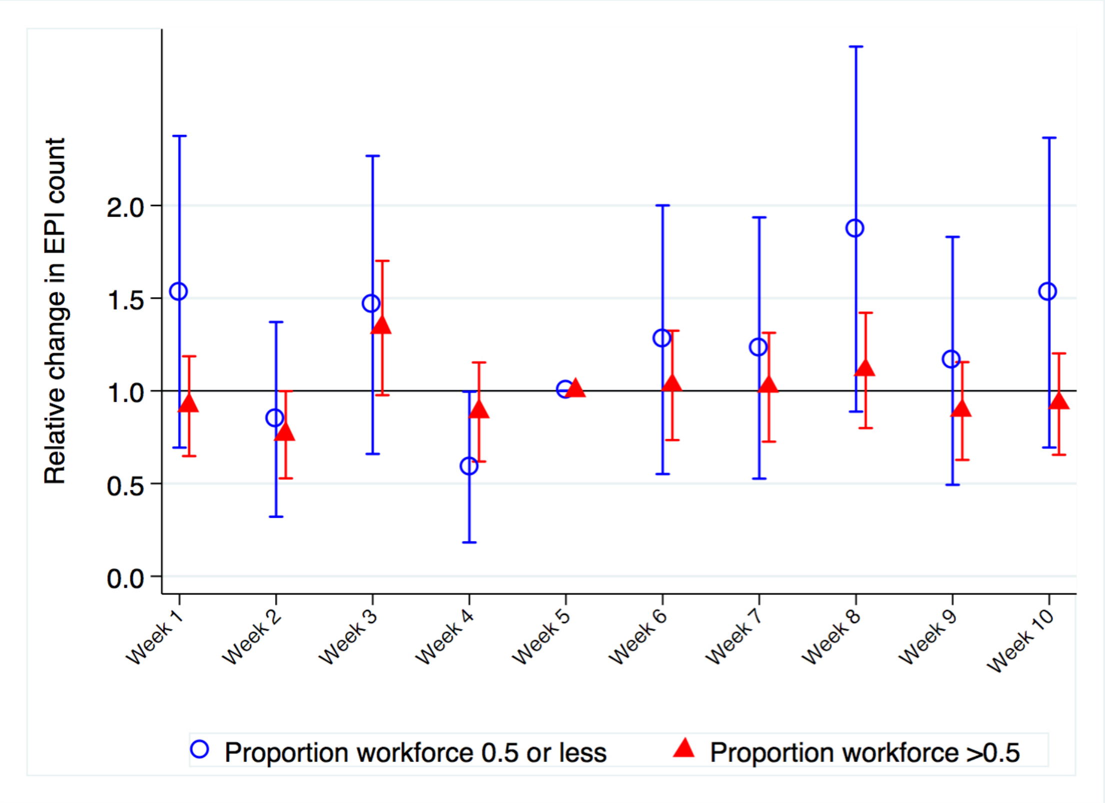
Relative change in EPI count
0.0
0.5
1.0
1.5
2.0
Week 1
Week 2
Week 3
Week 4
Week 5
Week 6
Week 7
Week 8
Week 9
Week 10
Proportion workforce 0.5 or less
Proportion workforce >0.5

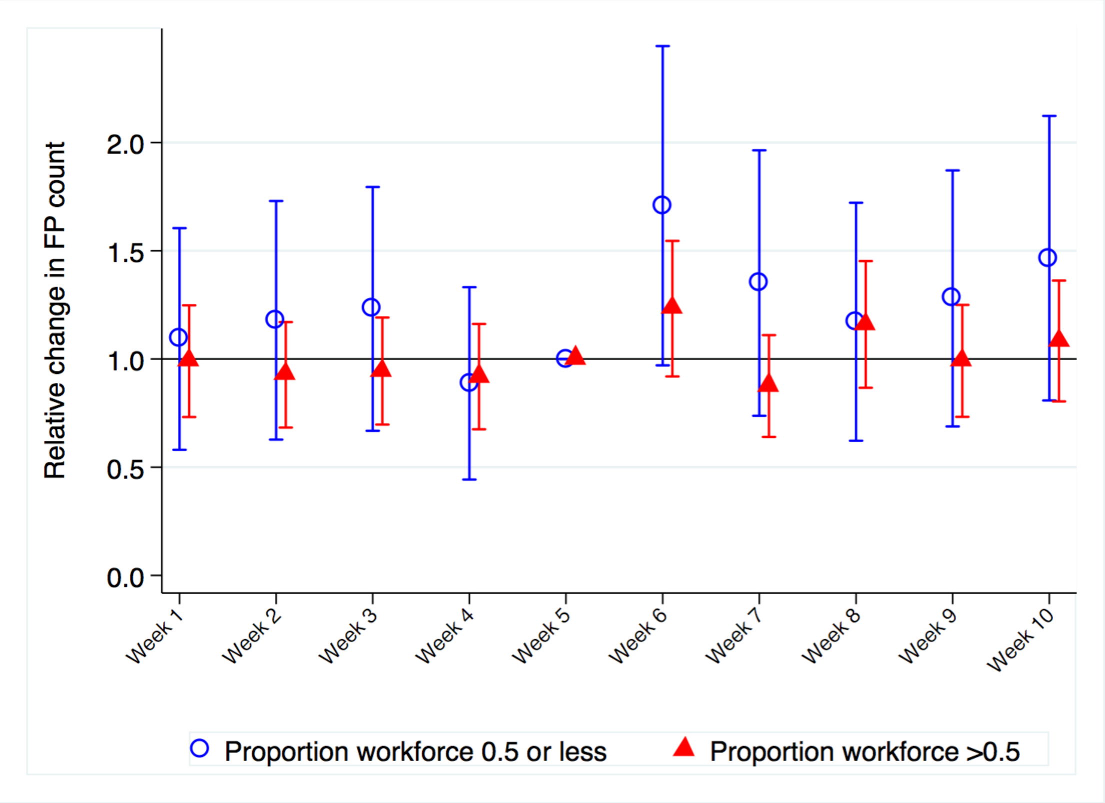
Relative change in FP count
0.0
0.5
1.0
1.5
2.0
Week 1
Week 2
Week 3
Week 4
Week 5
Week 6
Week 7
Week 8
Week 9
Week 10
Proportion workforce 0.5 or less
Proportion workforce >0.5
